# Strong selection signatures for Aleutian disease tolerance acting on novel candidate genes linked to immune and cellular responses in American mink (*Neogale vison*)

**DOI:** 10.1038/s41598-023-51039-7

**Published:** 2024-01-10

**Authors:** Seyed Milad Vahedi, Siavash Salek Ardestani, Mohammad Hossein Banabazi, K. Fraser Clark

**Affiliations:** 1 Department of Animal Science and Aquaculture, Dalhousie University, Bible Hill, NS B2N5E3 Canada; 2 Department of Animal Science, University of Zanjan, Zanjan, 4537138791 Zanjan Iran; 3 Department of Animal Breeding and Genetics (HGEN), Centre for Veterinary Medicine and Animal Science (VHC), Swedish University of Agricultural Sciences (SLU), 75007 Uppsala, Sweden; 4 Department of Biotechnology, Animal Science Research Institute of IRAN (ASRI),, Agricultural Research, Education & Extension Organization (AREEO), Karaj, 3146618361 Iran

**Keywords:** Genome evolution, Population genetics, Animal breeding, Agricultural genetics, Molecular evolution, Genome evolution

## Abstract

Aleutian disease (AD) is a multi-systemic infectious disease in American mink (*Neogale vison*) caused by Aleutian mink disease virus (AMDV). This study aimed to identify candidate regions and genes underlying selection for response against AMDV using whole-genome sequence (WGS) data. Three case–control selection signatures studies were conducted between animals (N = 85) producing high versus low antibody levels against AMDV, grouped by counter immunoelectrophoresis (CIEP) test and two enzyme-linked immunosorbent assays (ELISA). Within each study, selection signals were detected using fixation index (FST) and nucleotide diversity (θπ ratios), and validated by cross-population extended haplotype homozygosity (XP-EHH) test. Within- and between-studies overlapping results were then evaluated. Within-studies overlapping results indicated novel candidate genes related to immune and cellular responses (e.g., *TAP2*, *RAB32*), respiratory system function (e.g., *SPEF2*, *R3HCC1L*), and reproduction system function (e.g., *HSF2*, *CFAP206*) in other species. Between-studies overlapping results identified three large segments under strong selection pressure, including two on chromosome 1 (chr1:88,770–98,281 kb and chr1:114,133–120,473) and one on chromosome 6 (chr6:37,953–44,279 kb). Within regions with strong signals, we found novel candidate genes involved in immune and cellular responses (e.g., homologous MHC class II genes, *ITPR3*, *VPS52*) in other species. Our study brings new insights into candidate regions and genes controlling AD response.

## Introduction

Aleutian disease (AD), which is caused by the Aleutian mink disease virus (AMDV), is one of the most costly diseases affecting the American mink (*Neogale vison*) breeding industry^1,2^. Several mink-breeding countries have experienced AD outbreaks and losses, e.g., Canada in 1999–2002, 2007, 2012, and 2013^1,3–5^, Denmark in 2002, 2010–2011, and 2015–2016^6–8^, Spain in 2012–2014 and 2018^9^, Portugal in 2014^9^, and France in 2013^9^. Aleutian disease induces many issues in adult animals, including weight loss, lower fertility, and dropped pelt quality^10,11^. The disease can result in a > 90% mortality rate in kits, primarily due to induced acute interstitial pneumonia^12^. The antibody immune response plays a pivotal role in the pathogenesis of AMDV in kits and adults. A severe polyclonal hypergammaglobulinemia, referred to as “plasmacytosis”, is the hallmark of the progressive form of AD in adults^13^. In kits, the development of severe acute disease is associated with low or absent antibody titers paired with high levels of permissive viral replication^14^. Passive transmission of anti-AMDV antibodies can restrict viral replication and transcription and reduce pneumonia mortality and severity in kits^14^.

Aleutian disease outbreaks imposed substantial economic losses on mink farmers^1,3,5^. Meanwhile, eradication programs, using the gold-standard counter immunoelectrophoresis (CIEP) test for AD diagnosis, did not achieve satisfactory results mainly due to the high concentration of mink ranches, high seroprevalence of AD among wild mammals, and persistence of AMDV in the environment,^1,15–17^. Transmission of AMDV within and between farms with shared equipment, contaminated transport or feed vehicles, water streams, and potential mechanical vectors can also make the eradiction programs more challenging^18,19^. Efforts made to find an effective vaccine or a practical treatment have been unsuccessful. Therefore, selection for AD tolerance using enzyme-linked immunosorbent assays (ELISA) measuring antiviral antibodies against viral VP2 capsid protein (VP2 ELISA) and AMDVG viral antigen (AMDVG ELISA) has become the priority of mink farmers in some mink farming countries^4,20,21^. Animals producing low levels of anti-AMDV antibodies post-exposure to the virus demonstrate the “AD-tolerant” phenotype, possess an improved coordination between antibody and cellular response against AMDV, and are of interest for breeding in AD-positive herds^22–24^.

Advances in sequencing technologies provided an opportunity to investigate the patterns left behind in the genome associated with the natural or artificial selection process with a high resolution, mainly using single nucleotide polymorphisms (SNP)^25^. These patterns, so-called “signatures of selection”, can provide important information about the genes or genomic regions underlying selection, and contribute to a deeper understanding of genotype–phenotype relationships in the evolutionary context. The physical extent of such signatures, up and downstream of the functional variant, is a consequence of the so-called “selective sweep or hitchhiking effect”^26^. The characteristics of these genomic regions are: (i) the allele frequency is shifted towards extreme (high or low), (ii) there is an excess of homozygous genotypes, (iii) extended haplotypes exist with high frequency, and (iv) local population differentiation is extreme^27^. Several allele-frequency-based tests, e.g., fixation index (FST)^28^ and nucleotide diversity^29^, and extended haplotype homozygosity (EHH)-related statistics, e.g., cross-population extended haplotype homozygosity (XP-EHH)^30^, have been developed to detect genomic regions underlying selection. Moreover, the overlapping results of selection signature tests could be identified in order to improve the accuracy^31,32^.

To our knowledge, signatures of selection analysis for response against AMDV using whole-genome sequence (WGS) data have not been conducted in American mink. Here, we used WGS data to perform three case–control signatures of selection studies for host response against AMDV based on the results of CIEP, AMDVG ELISA, and VP2 ELISA immunoassays. Finally, the overlapping genomic regions and candidate genes among the three studies were identified to more precisely address genomic regions involved in response to AMDV. Annotated potential candidate genes in the respective chromosomal segments were studied in detail, including their functions and biological pathways. Our study could shed some light on the genetic architecture of American mink response to AMDV infection.

## Materials and methods

### Ethical statement

All procedures applied in this study were approved by the Dalhousie University Animal Care and Use Committee (certification nos. 2018-009, and 2019-012), and mink used were cared for according to the Code of Practice for the Care and Handling of Farmed Mink guidelines^33^. The study is reported in compliance with the ARRIVE guidelines.

### Population and phenotypic data

Animals were kept under standard farming conditions at the Canadian Centre for Fur Animal Research (CCFAR) at Dalhousie University, Faculty of Agriculture (Bible Hill, Canada). Animals were fed identical diets and had ad libitum access to diet and water. Phenotypic selection based on production traits, particularly pelt quality and reproductive performances, was the primary selection strategy in the CCFAR herd^34^. The CCFAR farm has likely experienced several outbreaks of AD, most recently in 2012 and 2013^1,4^. At the time of sampling, the seroprevalence of AD in the CCFAR herd was measured at 86.6% using the CIEP test. Considering the fact that AD can significantly impact mink pelt quality and fertility^10,11^, the two criteria by which animals were selected for the next generation in CCFAR farm, animals might have been indirectly selected for AD tolerance in this herd.

A total of 85 animals from the CCFAR farm (out of 905 animals) were selected for whole-genome sequencing in mid-November 2018. Animals were selected with respect to their color, sex, and the results of CIEP test, AMDVG ELISA, and VP2 ELISA. The maximum effort was performed to keep the highest level of phenotypic variation among the selected animals. Supplementary Table 1 presents the demographic data of animals included in this study.

Blood samples were taken for AD assessment in mid-November 2018 using toenail clipping. Three tests were performed on each animal’s sample: (i) CIEP test was performed at Animal Health Laboratory at the University of Guelph (Guelph, Canada), (ii) VP2 ELISA at the Nederlandse Federatie van Edelpelsdierenhouders (Wijchen, Netherlands), and (iii) AMDVG ELISA at Middleton Veterinary Services (Middleton, Canada). The CIEP test results were reported as positive or negative, representing detectable or undetectable levels of anti-AMDV antibodies. For AMDVG ELISA, the optical density (OD) results were reported as categories from 0 to 7, whereas for VP2 ELISA, the OD results were obtained as categories from 0 to 8. In both VP2 and AMDVG ELISA, lower categories represent a lower amount of anti-AMDV antibodies. Supplementary Table 2 presents the descriptive statistics of CIEP, VP2 ELISA, and AMDVG ELISA records of 85 animals included in this study.

### Sequencing, alignment, and variant calling

Two non-quality controlled variant call format (VCF) files previously generated by Genome Analysis Toolkit (GATK) and SAMtools/BCFtools software were provided for this study by Miar lab (https://miarlab.ca/), in which 15,102,221 and 13,468,882 SNPs from 85 American mink were present, respectively. Tissue sampling, deoxyribonucleic acid (DNA) extraction, sequencing, and trimming of sequences were conducted as described by Karimi et al.^35^. Briefly, genomic DNA was extracted from the tongue tissue using DNeasy Blood and Tissue Kit (Qiagen, Hilden, Germany) based on the kit protocol. DNA samples were then sequenced (100 bp pair-end reads) using the BGISEQ-500 platform at Beijing Genomics Institute (Guangdong, China). After sequencing, SOAPnuke software^36^ was used to remove adapters and low-quality reads. Then, high-quality reads were aligned against the latest American mink reference genome (GenBank accession no. GCA_020171115.1) using Burrows-Wheeler Aligner (BWA) 0.7.17 with default options^37^. The aligned files were converted to binary alignment map (BAM) format and sorted using SAMtools package version 1.11^38^. Potential duplicates were removed using the MarkDuplicates command tool of Picard (http://broadinstitute.github.io/picard/). The BAM files were then indexed by SAMtools software. Variant calling was performed with two pipelines, including: (i) mpileup module of SAMtools/BCFtools and (ii) GATK 4.1.7.0 variant calling pipeline (https://gatk.broadinstitute.org/). The GATK 4.1.7.0 HaplotypeCaller tool was used for variant calling to obtain high-quality results; however, the recalibration step was not performed due to the lack of a comprehensive database of known SNPs, i.e., reference VCF file.

### Quality control of variants

To increase the accuracy of SNP calling, the overlapping SNPs between two calling pipelines were extracted using a custom-made script in R software^39^. Then, using GATK 4.1.7.0, SNPs were filtered applying the phred-scaled quality score < 30.0, quality by depth < 2.0, phred scaled p-value using Fisher’s exact test to detect strand bias > 60.0, mapping quality < 40.0, strand odds ratio > 4.0, mapping quality rank sum test < -12.5 and read position rank sum test < -8.0 options. Remained SNPs were further filtered using VCFtools 0.1.16^40^ based on the following parameters: minor allele frequency < 0.01, max missing rate (for individuals) > 0.10, and Hardy–Weinberg p-value < 10^–7^. Moreover, only biallelic SNPs on autosomal chromosomes were kept for further analyses.

### Classification of animals to case and control groups

Due to long-term high seroprevalence of AD in the sampled farm, we assumed that all animals included in this study were exposed to AMDV. Subsequently, some animals developed detectable/high levels of anti-AMDV antibodies, but some were tolerant to the infection with undetectable/low levels of antiviral antibodies. Three phenotypic parameters of CIEP test, VP2 ELISA, and AMDVG ELISA results were used to divide animals into two extreme groups of animals producing high/detectable (cases) and low/undetectable (controls) anti-AMDV antibody levels. Therefore, three studies were designed to assess the signatures of selection for response against AMDV as follows:

1. signatures of selection study based on CIEP test,
2. signatures of selection study based on VP2 ELISA,
3. signatures of selection study based on AMDVG ELISA.

Table 1 represents the three case–control groups, the number of animals in each group, and the phenotypic criteria considered for the classification of animals. The number of overlapping individuals among case and control groups are depicted in Supplementary Figs. 1 and 2, respectively.

**Table 1:** Classification of animals into three case and control groups based on CIEP, AMDVG ELISA, and VP2 ELISA immunoassays.

Test	Case		Control	
	Number of animals	Description	Number of animals	Description
CIEP	48	Positive CIEP test, i.e., detectable antiviral antibody levels	31	Negative CIEP test, i.e., undetectable antiviral antibody levels
AMDVG ELISA	18	OD category of 8 in VP2 ELISA, i.e., very high antiviral antibody levels	31	OD category of 0 in VP2 ELISA, i.e., very low antiviral antibody levels
VP2 ELISA	10	OD category of 7 in AMDVG ELISA, i.e., very high antiviral antibody levels	35	OD category of 0 in AMDVG ELISA, i.e., very low antiviral antibody levels

### Signature of selection tests

In each study, three signatures of selection tests, including FST^28^, nucleotide diversity^29^, and XP-EHH^30^, were applied between case and control groups to detect regions underlying selection in the genome of American mink. FST and θπ ratios values were calculated using VCFtools 0.1.16^40^ for each SNP and averaged along 100 kb windows with a step size of 25 kb. The window size was determined based on the previous studies by Karimi et al.^31,35^. To normalize the FST values, Z-transformation was performed using scale command in R program^39^, and genomic windows harboring the top 1% Z-transformed FST values (Z_(FST)_) were identified. θπ ratios were then computed as θπ-case/θπ-control for all pairs of groups and then were log2-transformed (log_2(θπ ratios)_). Then, genomic windows harboring the top 1% absolute values were characterized.

XP-EHH values were calculated for each marker using selscan 2.0.0^41^ and averaged along 100 kb windows with a step size of 25 kb. Since selscan 2.0.0 could not manage missing genotypes, to keep a consistent number of markers with FST and nucleotide diversity analyses, missing genotypes were imputed, and genotypes were phased prior to XP-EHH test using Beagle software 5.2^42^. The unstandardized XP-EHH scores were transformed into a normal distribution using norm software provided in selscan package. Then, absolute normalized XP-EHH values were obtained using abs function in R software, and fitted to a normal distribution applying the robust linear model (rlm function) of the MASS 7.3 R package^43^ and model = rlm (abs(xpehh) ~ 1), where the abs(xpehh) object is a vector containing the absolute normalized XP-EHH values. The outputs of the fitted model, including mean and standard deviation, were used in pnorm R function to calculate the p-values of the XP-EHH statistics (lower.tail = FALSE, log.p = FALSE). Finally, to control multiple testing false discovery rate (FDR) among rejected null hypotheses, p-values were transformed to the corresponding q-values using qvalue R function and the Benjamini and Hochberg method^44^. Genomic windows with q-value < 0.05 were treated as significant regions detected by XP-EHH.

Regions overlapping within-studies, among the top 1% of genomic regions identified in Z_(FST)_ and log_2(θπ ratios)_ approaches and genomic regions with q-value < 0.05 in XP-EHH test, were considered as regions underlying putative selection in each study. Finally, between-studies overlapping genomic regions were identified. All circos plots were also created using the R package CMplot 3.6.2^45^. To show the overlaps within- and between-studies results, graphical visualization of American mink chromosomes was applied using the chromoMap 0.3.1 package^46^ in R software^39^.

### Candidate genes and enrichment analysis

The latest American mink genome assembly (GenBank accession no. GCA_020171115.1) and BEDTools 2.30.0 software^47^ were used for gene annotation of identified genomic regions. Based on the approach introduced by Karimi et al.^31^, gene ontology enrichment analysis was conducted based on the whole-genome reference list of domestic dog (*Canis lupus familiaris*), the closest species to American mink whose genome has been widely annotated^48^. PANTHER 16.0 (http://www.pantherdb.org/) and g:GOSt tool from g:Profiler (https://biit.cs.ut.ee/gprofiler/gost) were applied to determine Gene Ontology (GO) terms and biological pathways in Kyoto Encyclopedia of Genes and Genomes (KEGG)^49^ in which candidate genes were statistically over-represented. Overrepresented annotated genes were identified by Fisher’s exact test, and p-value adjusted by the FDR correction with < 0.05 considered as the threshold for significance.

## Results

A total of 12,639,732 overlapping SNPs were identified between variants called by GATK and SAMtools/BCFtools software. After quality control, 10,770,494 SNPs from 85 animals remained for further analysis. Within each study, a total of 92,791 genomic windows were scanned along the American mink genome to estimate Z_(FST)_ and log_2(θπ ratios)_ values between opposing case and control groups. The XP-EHH statistics were used to validate the detected signatures of selection by Z_(FST)_ and log_2(θπ ratios)_.

### Within-studies overlapping results

#### Signatures of selection study based on CIEP test

**Figure 1:**
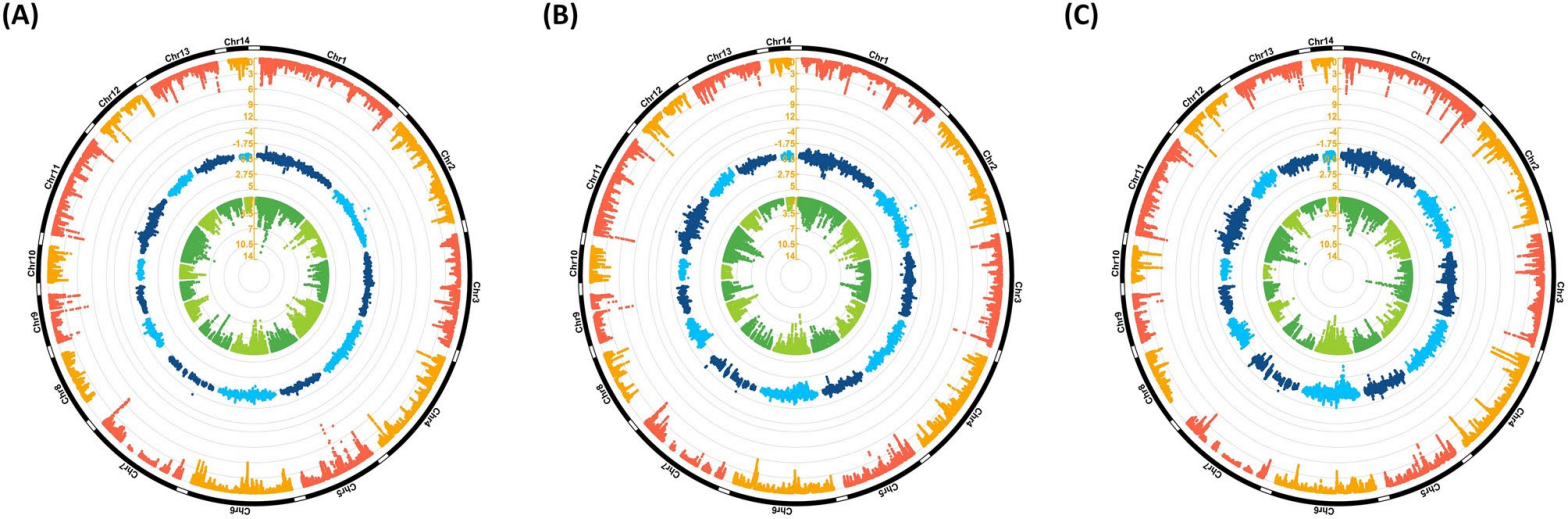
Red, blue, and green layers of circus plot show the distribution of (Z_(fst)_), log_2_(θπ ratios), and -log_10(p-value)_ of XP-EHH values, respectively. These values were calculated using a sliding window approach between animals grouped as cases and controls using CIEP test (**A**), VP2 ELISA (**B**), and AMDVG ELISA (**C**) records.

**Figure 2:**
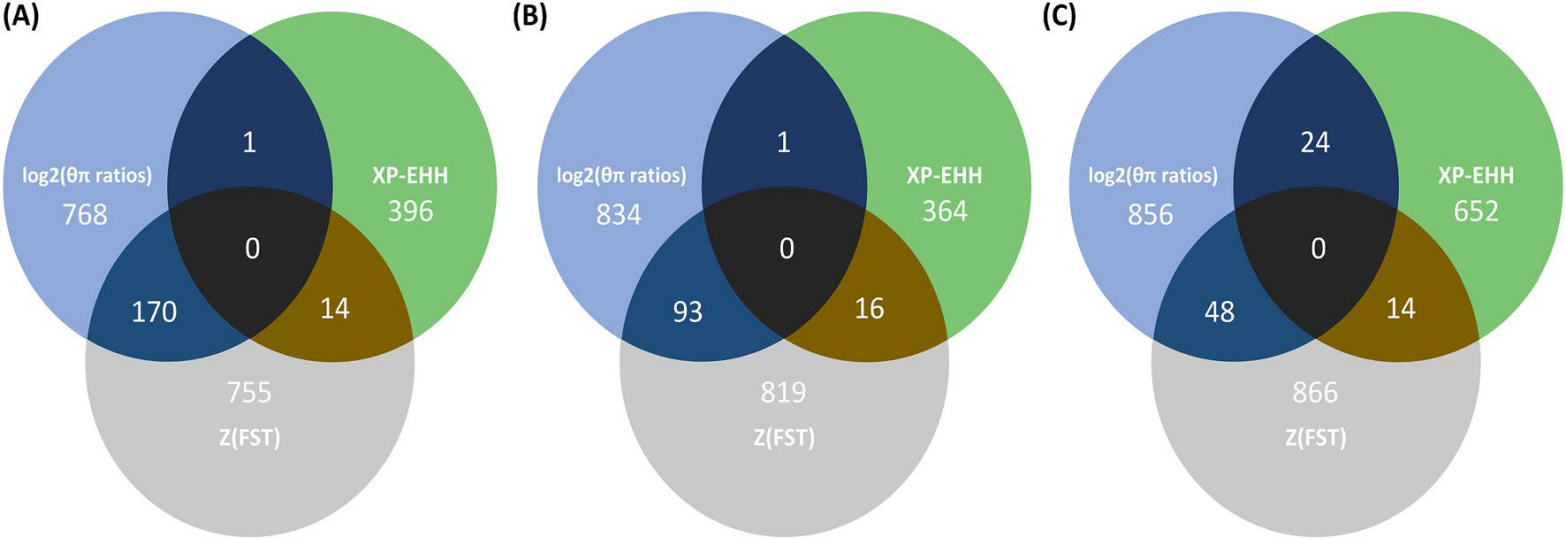
Venn diagrams of within-studies overlapping genomic windows, among top 1% regions obtained from Z_(FST)_ and log_2(θπ ratios)_ tests and the significant regions (q-value < 0.05) identified by XP-EHH test. Signatures of selection studies were applied between animals grouped as cases and controls using CIPE test (**A**), VP2 ELISA (**B**), and AMDVG ELISA (**C**) records.

Figure 1A and Supplementary Table 3 present the distribution of Z_(FST)_, log_2(θπ ratios)_, and -log_10(p-value)_ of XP-EHH statistics along the mink genome showing the potential signatures of selection signals based on the CIEP test results. Genomic windows harboring the top 1% Z_(FST)_ (n = 928) and log_2(θπ ratios)_ (n = 928) were identified. The top 1% Z_(FST)_ values ranged from 3.21 to 12.16, and the top 1% log_2(θπ ratios)_ ranged from 0.48 to 1.87. A total of 170 genomic regions were overlapped between the top 1% Z_(FST)_ and top 1% log_2(θπ ratios)_ regions (Fig. 2A). Gene annotation analysis of these regions identified a total number of 60 candidate genes potentially subjected to selection for response to AMDV infection (Supplementary Table 3). Table 2 presents candidate genes identified by different applied tests and studies that could be classified based on their potential functions in the host response to AMDV.

**Table 2:** Potential function of identified candidate genes underlying selection pressure in response to Aleutian mink disease virus (AMDV) based on their functions in other species.

Potential function	Genes symbol	Study^a^
Immune and cellular responses	*SLC35A1*, *RAB32*, *SERINC1*, *TNFRSF21*, *DST*, *ERAP2*, *AKAP9*, *RAP2B*, *ZMPSTE24*	CIEP
	*BAK1*, *ZBTB9*, *DAXX*, *LOXL4*, *LRRC15*, *USP38*, *BICD2*	VP2 ELISA
	*TAP2*, *FOXD2*, *ZNF484*, *OGN*	AMDVG ELISA
	*ITPR3*, *VPS52*	CIEP, VP2 ELISA
	*MYCBP2*, *BICD2*, *CADM2*	VP2 ELISA, AMDVG ELISA
Respiratory system	*SPEF2*, *R3HCC1L*	VP2 ELISA
	*CFAP206*^b^	CIEP
Reproductive system	*CFAP206*^b^, *HSF2*, *PEG10*, *ATR*	CIEP

These candidate genes were identified by three case–control signatures of selection studies based on CIEP, AMDVG ELISA, and VP2 ELISA immunoassays^a^, and only genes with potential function in the host response against Aleutian mink disease virus were listed. *CFAP206* is potentially involved in respiratory and reproductive systems function^b^.

XP-EHH test found 411 genomic windows with q-values < 0.05 (2.76 ≤ XP-EHH value ≤ 4.46); however, only 14 and 1 regions overlapped with Z_(FST)_ and log_2(θπ ratios)_ results, respectively, but no overlapped region with both tests were detected (Fig. 2A). Gene annotation of the overlapped regions with Z_(FST)_ and log_2(θπ ratios)_ identified six candidate genes potentially under selection pressure for response to AMDV infection (Supplementary Table 3). Out of six, five candidate genes were located within a segment on chromosome 2 (chr2:31,100–31,300 kb).

#### Signatures of selection study based on VP2 ELISA

The distribution of Z_(FST)_ and log_2(θπ ratios)_, as well as − log_10(p-value)_ of XP-EHH statistics based on the VP2 ELISA results were presented in Fig. 1B and Supplementary Table 4. We detected genomic windows harboring the top 1% Z_(FST)_ (n = 928) and log_2(θπ ratios)_ (n = 928). The top 1% of Z_(FST)_ values ranged from 3.26 to 8.80, and the top 1% log_2(θπ ratios)_ ranged from 0.77 to 2.71. A total of 93 genomic regions were overlapped between the top 1% Z_(FST)_ and top 1% log_2(θπ ratios)_ regions (Fig. 2B). A total number of 44 candidate genes were identified in gene annotation analysis of these regions. A complete list of candidate regions and their positions is provided in Supplementary Table 4.

XP-EHH test identified 381 genomic windows with q-values < 0.05 (2.76 ≤ XP-EHH value ≤ 4.05); however, only 16 and 1 regions overlapped with Z_(FST)_ and log_2(θπ ratios)_ results, respectively, and no overlapped region with both tests were detected (Fig. 2B). Gene annotation of the overlapped regions with Z_(FST)_ and log_2(θπ ratios)_ resulted in four candidate genes (Supplementary Table 4). Out of four, two genes were located on chromosome 2 (chr2:237,800–237,900 kb), and two were on chromosome 9 (chr9:97,700–97,800 kb).

#### Signatures of selection study based on AMDVG ELISA

The distribution of Z_(FST)_ and log_2(θπ ratios),_ and -log10(p-value) of XP-EHH statistics based on AMDVG ELISA records were shown in Fig. 1C and Supplementary Table 5. Genomic windows harboring the top 1% Z_(FST)_ (n = 928) and log_2(θπ ratios)_ (n = 928) were identified. The top 1% Z_(FST)_ values ranged from 3.08 to 10.77, and the top 1% log_2(θπ ratios)_ ranged from 0.98 to 4.40. A total of 48 genomic regions were overlapped between the top 1% Z_(FST)_ and top 1% log_2(θπ ratios)_ regions (Fig. 2C). Gene annotation analysis of these regions identified a total number of 23 candidate genes potentially subjected to selection for response to AMDV infection (Supplementary Table 5).

XP-EHH test detected 690 genomic windows with q-value < 0.05 (2.61 ≤ XP-EHH value ≤ 4.06); among which 14 and 24 regions overlapped with Z_(FST)_ and log_2(θπ ratios)_ results, respectively. Similar to CIEP and VP2 ELISA studies, no overlapping regions with both tests were detected (Fig. 2C). Gene annotation of the overlapped regions with Z_(FST)_ and log_2(θπ ratios)_ identified 16 candidate genes potentially under selection pressure for response to AMDV infection (Supplementary Table 5). Out of 16, eight candidate genes were located on a segment chromosome 9 (chr9:97,400–97,700 kb).

### Between-studies overlapping results

**Figure 3:**
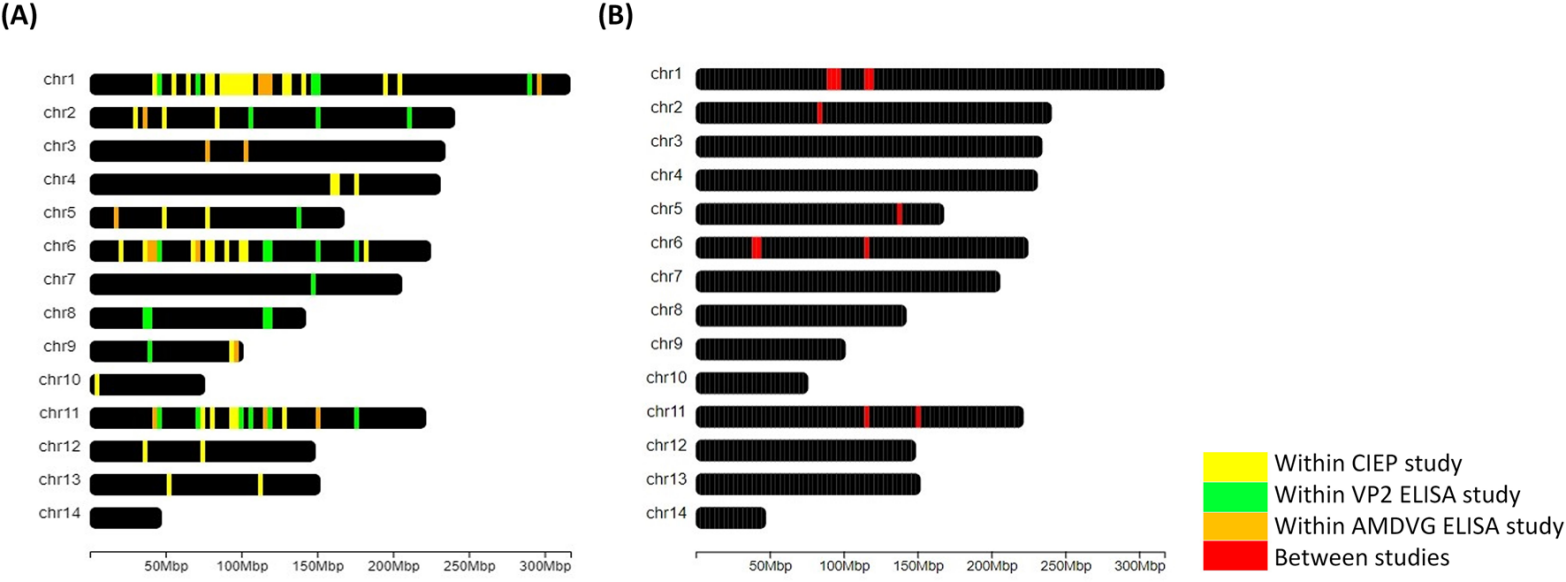
Graphical visualization of the American mink chromosomes depicting overlapping within- (**A**) and between-studies (**B**) genomic regions underlying selection pressure.

**Figure 4:**
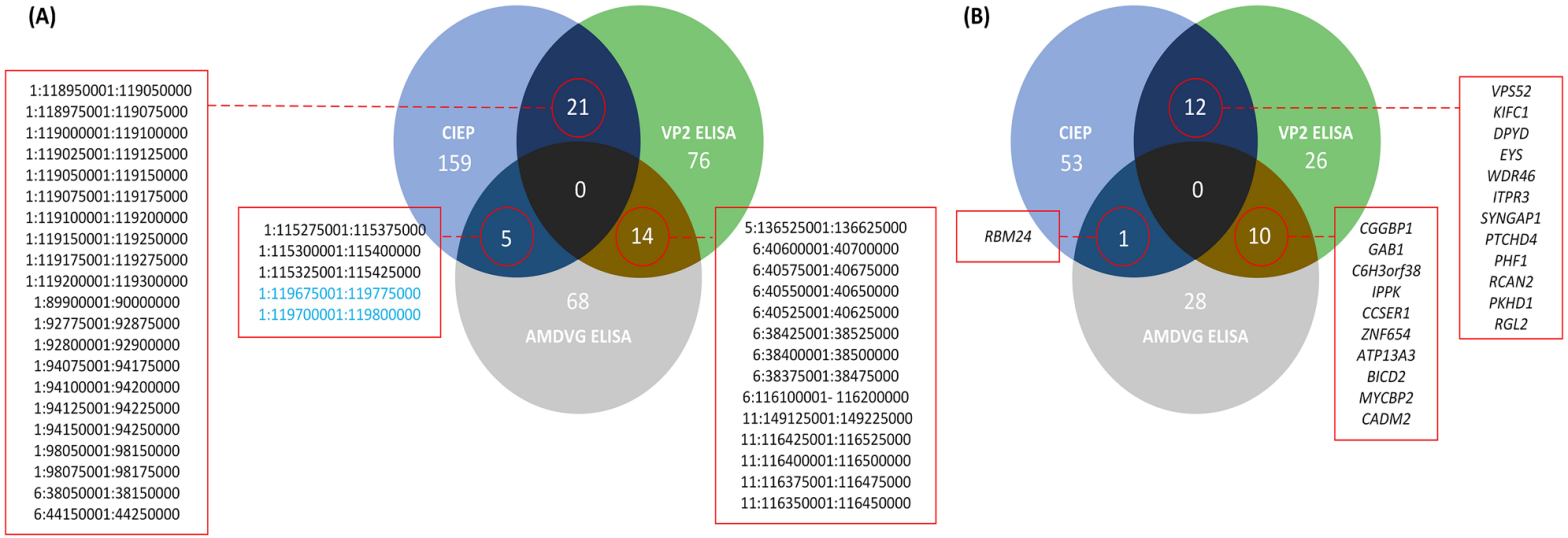
Venn diagram of between-studies overlapping genomic regions (**A**) and candidate genes (**B**). The genomic regions highlighted in blue color represent segments on chromosome 1 overlapping with homologous human (HLA) and rabbit (RLA) leukocyte antigen genes loci (chr1:119,357–119,996 kb).

The total number of identified candidate regions was 342, varying from 86 for AMDVG ELISA to 189 for the CIEP study. Gene annotation analysis of these regions identified 130 candidate genes, with a maximum of 66 genes in CIEP study and a minimum of 39 genes in AMDVG ELISA study (Table 3). We also found 40 strong selection signals overlapping between studies. These genomic regions, which were located on chromosomes 1, 2, 5, 6, and 11, annotated 23 candidate genes (Fig. 3). Figure 4 shows the overlaps among the genomic windows and candidate genes identified in conducted studies. Moreover, Table 4 presents the studies and tests by which each candidate gene was identified.

**Table 3:** Number of candidate regions and genes detected by signatures of selection studies based on CIEP test, AMDVG ELISA, and VP2 ELISA records.

Grouping criteria	Number of candidate regions	Number of candidate genes
CIEP	189	66
VP2 ELISA	110	48
AMDVG ELISA	86	39
Overlapped^a^	40	23
Total^b^	342	130

Overlapped candidate regions/genes^b^ represents the number of between-studies overlapping regions/genes. Total number of candidate regions/genes^a^ does not include duplicates.

**Table 4:** Candidate genes identified by between-studies overlapping genomic regions.

Chromosome	Start position (bp)	End position (bp)	Gene ID	Study			Test		
				CIEP	VP2 ELISA	AMDVG ELISA	Z_(FST)_	log_2(θπ ratios)_	XP-EHH
1	92,538,026	92,807,355	*RCAN2*	×	×	−	×	×	−
1	94,047,591	94,230,349	*PTCHD4*	×	×	−	×	×	−
1	97,738,076	98,213,846	*PKHD1*	×	×	−	×	×	−
1	105,941,472	107,593,901	*EYS*	×	×	−	×	×	−
1	118,914,596	118,980,320	*ITPR3*	×	×	−	×	×	−
1	119,108,583	119,140,172	*SYNGAP1*	×	×	−	×	×	−
1	119,144,037	119,149,122	*PHF1*	×	×	−	×	×	−
1	119,150,291	119,163,372	*KIFC1*	×	×	−	×	×	−
1	119,225,004	119,232,375	*RGL2*	×	×	−	×	×	−
1	119,234,730	119,243,361	*WDR46*	×	×	−	×	×	−
1	119,252,887	119,270,337	*VPS52*	×	×	−	×	×	−
1	139,672,472	139,682,922	*RBM24*	×	−	×	×	×	−
2	83,029,941	83,871,891	*DPYD*	×	×	−	×	×	−
5	136,533,697	136,816,788	*MYCBP2*	−	×	×	×	×	−
6	37,748,501	38,827,099	*CADM2*	−	×	×	×	×	−
6	40,554,251	40,558,056	*CGGBP1*	−	×	×	×	×	−
6	40,558,281	40,640,279	*ZNF654*	−	×	×	×	×	−
6	40,644,230	40,654,566	*C6H3orf38*	−	×	×	×	×	−
6	116,089,406	116,181,494	*ATP13A3*	−	×	×	×	×	−
9	97,712,221	97,758,164	*IPPK*	−	×	×	−	×	×
9	97,789,108	97,832,330	*BICD2*	−	×	×	−	×	×
11	115,732,693	116,968,169	*CCSER1*	−	×	×	×	×	−
11	149,036,912	149,163,525	*GAB1*	−	×	×	×	×	−

Within each study, three selection signatures tests of Z_(FST)_, log_2(θπ ratios)_, and XP-EHH were applied.

### Gene enrichment

All 130 candidate genes detected by our selection signature studies were applied to gene classification and ontology enrichment analyses. Identified candidate genes in this study were mainly involved in the binding activity (46%). Supplementary Fig. 3 presents a pie chart of the molecular function classification of the candidate genes underlying selection for response against AMDV. Gene functional enrichment analysis using g:GOSt and PANTHER tools detected 34 and one significantly overrepresented terms, respectively (Supplementary Table 6). The most significant identified term was protein binding molecular function (GO term = GO:0005515; FDR adjusted p-value = 0.000). We also identified several significantly overrepresented terms related to the heart function (Supplementary Table 6).

## Discussion

Aleutian disease was first detected in Canada in the late 1950s^50^; however, several outbreaks have been reported in mink farms across Atlantic Canada, e.g., Nova Scotia in 1999–2002^5^, 2012^1,4^, 2013^1,4^, and Newfoundland in 2007^3^, where the mink farming practice is common. It is believed that animals have been intensively under natural selection for several generations due to fatal AMDV infection. Our study prepared the first map of signatures of selection for response to ADMV infection at the chromosome level using WGS data and based on the new gene annotation after the release of the American mink genome assembly at the chromosomal level by Karimi et al.^51^. WGS data has already been used to detect selection signals in other domesticated animals, such as pigs^52^ and sheep^53^. Karimi et al.^31^ conducted a signatures of selection study between the extreme subgroups of a single population of American mink inoculated with AMDV. Moreover, the same approach was applied to other traits in livestock animals, such as fat deposition in sheep^54^, feather pecking in chickens^55^, climate adaptation^56^ and resistance to mastitis^57^ in cattle, and parasite resistance in goats^58^.

### Within-studies overlapping regions and genes

#### *Regions and genes detected by Z*_*(FST)*_* and log*_*2(θπ ratios)*_

Our study could successfully address genomic regions potentially involved in response against AMDV in American mink. Most of the within-studies overlapping genomic regions identified by CIEP and VP2 ELISA studies were located on chromosome 1 (Supplementary Tables 3 and 4). Meanwhile, AMDVG ELISA study indicated some signals on chromosomes 3 and 9 (Supplementary Table 5). Gene annotation study of these regions found several genes involved in immunity and cellular response to pathogens (Table 2). For example, *TAP2* is a major histocompatibility complex (MHC) gene whose products construct a transporter molecule that contributes to antigen processing^59^. The role of this gene in the innate immune response to several viral infections in cattle^60^ and humans^61^ has been reported. Saravanan et al.^60^ revealed that *TAP2* is critical in cattle’s robust innate immune response against foot-and-mouth disease virus infection. In humans, the induction of *TAP2* has an important implication for the immune response to Epstein-Barr^61^ and hepatitis C virus^62^. Therefore, *TAP2* might be involved in the antigen processing of AMDV, the intensity of antibody response, and the pathogenicity of AMDV could be boosted through interruption of *TAP2* function by viral proteins.

Another candidate gene which is involved in immune and cellular responses was *RAB32*. This gene encodes an A-kinase anchoring protein and is involved in mitochondrial dynamics^63^. It has been recently found that *RAB32* promotes the proliferation of effector CD8 + T cells in response to the challenges with cellular antigens *in-vivo*^64^. With regards to AD, cytotoxic T cells (CD8+) functions and their interferon signaling pathway play critical roles in the restriction of AMDV persistence and replication^65,66^. Another candidate gene is *SERINC1* encoding Serine Incorporator 1, which facilitates the synthesis of serine-derived lipids^67^. Disruption of *SERINC1* can lead to the failure of macrophage function alteration and lymphocyte proliferation^68^. Concerning AD, unrestricted B cell proliferation and antibody production^69^, as well as the malfunction of macrophages in the antibody-dependent enhancement process^70^ are the main features of the progressive form of the disease in adults. We also identified *DAXX* gene encoding a signaling protein whose overexpression enhances Fas-mediated apoptosis and activates the Jun N-terminal kinase (JNK) pathway^71^. It has been reported that in immediate-early viral gene expression of herpes simplex virus 1 (HSV-1), human cytomegalovirus (HCMV), human Epstein-Barr virus (EBV), and Kaposi’s sarcoma-associated herpesvirus (KSHV), several virion proteins may counteract intrinsic immune mechanisms mediated by the promyelocytic leukemia protein (PML) nuclear body (PML NB)-associated cellular factors such as *DAXX*^72^. More targeted studies are necessary to confirm the role of *DAXX* in AMDV pathogenesis.

The gene annotation analysis of genomic regions under selection found some candidate genes involved in respiratory system functions (Table 2). *SPEF2* has been postulated to play an essential role in cilia assembly^73^. AD with fatal respiratory distress and fulminant interstitial pneumonia mainly occurs in American mink kits due to permissive and cytopathic replication of the virus in the lung type II pneumocytes^12,13^. Clearance by ciliary motility is an essential response to respiratory pathogens, and defects in ciliary motility can result in a severe response to pulmonary infection^74^. Some *SPEF2* alleles might contribute to motility defects in mink respiratory cilia leading to more susceptibility to AMDV-induced pulmonary lesions. Another identified candidate gene was *R3HCC1L* encoding a coiled-coil domain-containing protein. Recently, variants in the region of this gene have been found to be associated with human susceptibility to infectious pneumonia^75^. However, the molecular function of these genes is still unclear in American mink, and more studies are necessary to clarify *R3HCC1L* role in the pathogenesis of AMDV.

We identified some candidate genes linked with reproductive system function (Table 2). For example, *HSF2* is a member of heat shock transcription factors family that are significant transactivators of heat shock protein genes in response to stress, and they are involved in embryonic development and spermatogenesis^76^. A study by Wang et al.^77^ showed that *HSF2* has a significant function in controlling the expression of genes essential for embryonic development. Notably, the risk of reproductive failure, including conception rate, size, weight of litter at birth, and neonatal mortality, is much higher than normal in AD-positive dams^11^. Therefore, we suggest more studies to investigate the role *HSF2* in dams’ infertility caused by AMDV. Another candidate gene was *CFAP206* which encodes Cilia and Flagella Associated Protein 206 localized to the basal body and the motile cilia axoneme. This protein is required for sperm motility and mucociliary clearance of the airways^78^. Recent studies showed impaired *CFAP206* might result in male infertility and dysfunction of mucociliary clearance of the airways^78,79^. It is noteworthy that AMDV in kits and adults can lead to acute interstitial pneumonia and infertility, respectively^80,81^. Therefore, *CFAP206* may play a key role in the determination of multiple phenotypes of response to AMDV such as infertility of adults and pneumonia in kits.

#### Regions and genes validated by XP-EHH test

We identified some genomic regions on chromosomes 2, 3, 5, and 9 that were validated by XP-EHH test (Supplementary Tables 3–5), representing regions that are more likely involved in response against AMDV. Genomic regions validated by XP-EHH test annotated several candidate genes involved in immune and cellular responses (Table 2). Among these genes, the critical role of *ZMPSTE24* in response to viral infections has been well-documented. *ZMPSTE24* encodes a transmembrane metalloprotease whose catalytic activity is essential for processing lamin A on the inner nuclear membrane and clearing clogged translocons on the endoplasmic reticulum. *ZMPSTE24* is a component of a common antiviral pathway that is associated with interferon-induced transmembrane proteins^82^. In a study conducted by Fu et al.^83^, *ZMPSTE24* was reported as a virus-specific effector that restricts multiple RNA and DNA viruses, including influenza A, Zika, Ebola, Sindbis, vesicular stomatitis, cowpox, and vaccinia. *ZMPSTE24* can combine with the interferon-induced transmembrane protein (IFITM) family, which eventually impedes viral entry^83^. Further studies on this gene and its role in the restriction of AMDV internalization are suggested. We also identified *FOXD2* gene contributing to the sensitivity to cAMP in T Lymphocytes through the regulation of cAMP-dependent Protein Kinase Riα^84^. This protein sets the threshold for cAMP-mediated negative modulation of T cells activation^84^. Therefore, this gene might be involved in the regulation of T cells responses against AMDV.

### Between-studies overlapping regions and genes

A total of 40 overlapping between-studies genomic regions were detected (Table 3), addressing strong selection signals for response against AMDV. We detected three long regions under intensive selection, including two segments on chromosome 1 (chr1:88,770–98,281 kb and chr1:114,133–120,473 kb) and one segment on chromosome 6 (chr6:37,953–44,279 kb) (Figs. 3), suggesting that these regions might have some roles in controlling quality of animals’ response against AMDV. More than half of the between-studies overlapping regions were identified between two studies based on CIEP and VP2 ELISA results. The reason could be that the control groups of these two studies were more comparable compared with AMDVG ELISA’s (Supplementary Fig. 2).

Fifteen genomic windows under intensive selection were located within the second large segment on chromosome 1 (chr1:114,133–120,473 kb). This segment harbors homologous human (HLA) and rabbit (RLA) leukocyte antigen genes (chr1:119,357–119,996 kb) in the last American mink genome assembly (GenBank accession no. GCA_020171115.1). These homologous genes included LOC122911007, LOC122911034, LOC122911086, LOC122911160, LOC122911197, LOC122911212, LOC122911237 encoding homologous proteins to DP beta 1 chain (DPB1)-like of HLA class II, DP beta 1 chain (DPB1)-like of RLA class II, DO alpha chain (DOA) of HLA class II, DM alpha chain (DMA) of HLA class II, DM beta chain (DMB) of HLA class II, DO beta chain (DOB) of HLA class II, DQ beta 1 (DQB1) chain of HLA class II, DQ alpha 2 chain (DQA2)-like, respectively.

MHC Class II molecules are typically found only on antigen-presenting cells such as dendritic cells, mononuclear phagocytes, some endothelial cells, thymic epithelial cells, and B cells^85^. Antigen-presenting cells display a range of peptides for recognition by the T-cell receptors of CD4 + T helper cells using MHC class II molecules^86^. Therefore, MHC class II molecules are necessary for effective adaptive immune responses against viral infections. MHC class II molecules consist of two homogenous peptides of alfa and beta chains, and the sub-designation of alpha 1, alpha 2, beta 1, and beta 2 refers to the separate domains within the leukocyte antigen gene^87^. Typically, the chains’ alpha 1 and beta 1 regions merge to form a peptide-binding domain, whereas the alpha 2 and beta 2 regions construct an immunoglobulin-like domain together^88^. Two effective strategies of viruses to escape detection by CD4 + T cells are: (a) inhibiting the MHC class II antigen presentation pathway by affecting the stability or intracellular sorting of class II proteins; (b) inhibiting the expression of MHC class II genes by blocking interferon-γ signal transduction and expression of the MHC class II transactivator^89^. To the best of our knowledge, the role of MHC class II genes in American mink response against pathogens has not been studied yet; however, the significant role of the MHC class II in human immune response against parvovirus B19, the only autonomous parvovirus known to infect humans, has been well documented^90–92^. Due to the central role of MHC class II genes in the vertebrate immune system, it is presumed that ADMV-driven selection may act on MHC class II genes. Therefore, some combinations of MHC class II genes might significantly influence AMDV susceptibility or tolerance. More attention must be given to the role of MHC class II genes cluster in the quality of immune response against AMDV in American mink.

A total of 23 between-studies overlapping candidate genes were identified under strong selection pressure (Table 4). Among them, we identified five genes of *ITPR3*, *VPS52*, *MYCBP2*, 5 *BICD2*, and *CADM2* involved in immune and cellular responses to pathogens. *ITPR3* is a receptor for inositol 1,4,5-trisphosphate (IP3), a second messenger that mediates intracellular calcium release. IP3 can be phosphorylated by inositol 1,4,5-trisphosphate 3-kinase C and downregulate the T cell signaling pathway by regulating Ca+/nuclear factors^93^. In addition, the *ITPR3* gene activates T lymphocyte apoptosis^94^, and T cells significantly restrict AMDV infection in mink^23^. Another identified candidate gene in this study was *VPS52*, a GARP complex gene subunit. It has been demonstrated that *VPS52* is required for extracellular monkeypox^95^ and vaccinia^96^ virus formation. Recently, this gene was also identified as a candidate gene affecting resistance to Tilapia lake virus in farmed Nile tilapia^97^. Therefore, we suggest more investigations on the role of this gene on AMDV resistance in American mink.

### Gene enrichment

Gene classification indicated that most identified genes were involved in the binding activity. Moreover, gene enrichment analysis of candidate genes under selection identified several significantly overrepresented terms related to protein binding. AMDV pathogenicity and host immune response against the virus include a wide range of binding activities^98,99^. Therefore, host response against AMDV might be mediated by genes controlling binding activities. Gene enrichment analysis also found several overrepresented terms associated with heart functions, such as regulation of ventricular cardiac muscle cell membrane repolarization, cardiac conduction, cardiac muscle cell contraction regulation. Macroscopic enlarged hearts with microscopic lesions and widespread plasma cell infiltration were previously reported in AMDV-infected mink^100,101^. Therefore, the adaptive response of heart cells to the stress induced by AMDV infection might be controlled by genes involved in heart functions.

### Limitations and future works

In this study, our results were limited to genomic regions under selection pressure in response to AMDV, but it is still unknown which regions contribute significantly to AD tolerance. To address genomic regions underlying AD tolerance, we suggest genome-wide association studies, including CIEP-positive animals recorded for VP2 and AMDVG ELISA. Also, our study was limited to the small population of animals in one AD-positive population; however, using larger sample sizes can improve the power of genome-wide studies.

We detected the most significant selection signature signals on a segment on chromosome 1, where homologous MHC class II genes were located. We expect that the identified region might have a crucial role in determining the quality of American mink immune response in AMDV infections, which is economically favorable for mink industry. Therefore, more targeted studies on this segment to investigate its function are highly recommended.

## Conclusion

Our study demonstrated the first map of selection signals for response to ADMV infection at the chromosome scale using WGS data. We detected 342 candidate regions and 130 candidate genes associated with the anti-AMDV response using signatures of selection tests of Z_(FST)_, log_2(θπ ratios)_, and XP-EHH based on the records of CIEP test, VP2 ELISA, and AMDVG ELISA. Between-studies overlapping results were eventually reported as genomic regions more likely subjected to the selection process for response against AMDV infection, including 40 genomic regions and 23 candidate genes. We introduced a series of novel candidate genes that might be involved in the host response or targeted by the virus, including genes contributing to the immune and cellular responses and some related to the functions of respiratory and reproductive systems. Our findings provide new genetic insight into the process of response to AMDV infection. The results confirm the complexity of the genetic mechanisms underlying host response against AMDV while suggesting that this response might be partly under the control of MHC class II genes. Our findings help better understand the animal’s response to AMDV and the pathogenesis of the virus, which can be further applied to developing an effective vaccine or treatment or improving mink breeding programs.

### Supplementary Information

Supplementary Information.

## Data Availability

The phenotype and genotype data supporting this study are owned by the mink industry. The generated data are confidential and protected as intellectual property. As a result, the data analyzed in this study are not publicly available. Any interested parties would need to obtain consent from the mink industry’s researcher at Dalhousie University, Dr. Younes Miar (miar@dal.ca), prior to contacting the corresponding authors. Supplementary Tables 3, 4, and 5 contain the complete list of identified candidate genes that are not shown in the main text. All generated scripts for the analyses are available at: https://github.com/smiledv/minkad.
