# Publisher Correction: Strong selection signatures for Aleutian disease tolerance acting on novel candidate genes linked to immune and cellular responses in American mink (*Neogale vison*)

**DOI:** 10.1038/s41598-024-57764-x

**Published:** 2024-04-03

**Authors:** Seyed Milad Vahedi, Siavash Salek Ardestani, Mohammad Hossein Banabazi, K. Fraser Clark

**Affiliations:** 1 Department of Animal Science and Aquaculture, Dalhousie University, Bible Hill, NS B2N5E3 Canada; 2 Department of Animal Science, University of Zanjan, Zanjan, 4537138791 Zanjan Iran; 3 Department of Animal Breeding and Genetics (HGEN), Centre for Veterinary Medicine and Animal Science (VHC), Swedish University of Agricultural Sciences (SLU), 75007 Uppsala, Sweden; 4 Department of Biotechnology, Animal Science Research Institute of IRAN (ASRI), Agricultural Research, Education & Extension Organization (AREEO), Karaj, 3146618361 Iran

Correction to: *Scientific Reports* 10.1038/s41598-023-51039-7, published online 10 January 2024

The original version of this Article contained an error in the caption of Figure 1.

“HYPERLINK "sps:id::fig1||locator::gr1||MediaObject::0"Red, blue, and green layers of circus plot show the distribution of (Z_(fst)_), log2(θπ ratios), and -log_10(p-value)_ of XP-EHH values, respectively. These values were calculated using a sliding window approach between animals grouped as cases and controls using CIEP test (**A**), VP2 ELISA (**B**), and AMDVG ELISA (**C**) records.”

now reads:

“Red, blue, and green layers of circus plot show the distribution of (Z_(fst)_), log_2_(θπ ratios), and -log_10(p-value)_ of XP-EHH values, respectively. These values were calculated using a sliding window approach between animals grouped as cases and controls using CIEP test (**A**), VP2 ELISA (**B**), and AMDVG ELISA (**C**) records.”

The original Article has been corrected.

