# Supplementary material for: Strong selection signatures for Aleutian disease tolerance acting on novel candidate genes linked to immune and cellular responses in American mink (Neogale vison)
Source: Sci Rep. 2024 Jan 10;14:1035. doi: 10.1038/s41598-023-51039-7 (PMC10781757; doi:10.1038/s41598-023-51039-7)
Supplement: Supplementary file 1 — Supplementary Information. [file 41598_2023_51039_MOESM1_ESM.docx]

**Supplementary tables**

**Supplementary Table 1.** Demographic information of animals included in this study.

|  | | Color | | | | | Total |
| --- | --- | --- | --- | --- | --- | --- | --- |
|  |  | Dark | Demi | Mahogany | Pastel | Stardust |  |
| Sex | Female | 7  (8.2%) | 22  (25.9%) | 13  (15.3%) | 5  (5.9%) | 3  (3.5%) | 50 (58.8%) |
|  | Male | 9  (10.6%) | 10  (11.8%) | 7  (8.2%) | 5  (5.9%) | 4  (4.7%) | 35 (41.2%) |
| Total | | 16  (18.8%) | 32  (37.7%) | 20  (23.5%) | 10  (11.8%) | 7  (8.2%) | 85 (100.0%) |

**Supplementary Table 2.** Descriptive statistics for CIEP, VP2 ELISA, and AMDVG ELISA records in animals included in this study.

| Test | N^1^ | CIEP+^2^ | CIEP-^2^ | VP2 cat. 8^3^ | VP2 cat. 0^3^ | AMDVG cat. 7^4^ | AMDVG cat. 0^4^ | Mean^1^ | SD^1^ | Min^1^ | Max^1^ | CV (%)^1^ |
| --- | --- | --- | --- | --- | --- | --- | --- | --- | --- | --- | --- | --- |
| CIEP | 79 | 48 | 31 | - | - | - | - | 0.61 | 0.49 | 0 | 1 | 80.33 |
| VP2 ELISA | 85 | - | - | 18 | 31 | - | - | 3.82 | 2.78 | 0 | 8 | 72.77 |
| AMDVG ELISA | 85 | - | - | - | - | 10 | 35 | 2.79 | 3.40 | 0 | 7 | 121.86 |

^1^N: number of records, mean: arithmetic mean, including the percentage of positive animals based on CIEP test and the average of VP2 and AMDVG ELISA optical density values, SD: standard deviation, Min: minimum, Max: maximum, CV (%): coefficient of variation percentage.

^2^CIPE+: number of animals with CIEP positive record; CIEP-: number of animals with CIEP negative record.

^3^VP2 cat. 8: number of animals classified in category 8 in VP2 ELISA test; VP2 ELISA cat. 0: number of animals classified in category 0 in VP2 ELISA test.

^4^AMDVG cat. 7: number of animals classified in category 7 in ADMVG ELISA test; AMDVG cat. 0: number of animals classified in category 0 in AMDVG ELISA test.

**Supplementary Table 3.** Identified candidate genes located within the putatively selected regions overlapping between tests within CIEP study.

| Chr^a^ | Start position (bp^b^) | End position (bp) | Gene ID | Z_(FST)_ | log_2(θπ ratios)_ | XP-EHH |
| --- | --- | --- | --- | --- | --- | --- |
| 1 | 42,756,498 | 42,780,882 | *SLC35A1* | ×^c^ | × | -^d^ |
| 1 | 42,787,791 | 42,844,995 | *CFAP206* | × | × | - |
| 1 | 42,876,448 | 42,893,869 | *C1H6orf163* | × | × | - |
| 1 | 42,935,707 | 42,948,189 | *GJB7* | × | × | - |
| 1 | 56,358,569 | 56,377,670 | *RAB32* | × | × | - |
| 1 | 77,770,526 | 77,788,115 | *SMPDL3A* | × | × | - |
| 1 | 77,829,681 | 77,933,740 | *PKIB* | × | × | - |
| 1 | 78,022,879 | 78,057,252 | *SERINC1* | × | × | - |
| 1 | 78,059,083 | 78,094,914 | *HSF2* | × | × | - |
| 1 | 81,473,536 | 81,484,315 | *PLN* | × | × | - |
| 1 | 81,404,474 | 81,594,095 | *CEP85L* | × | × | - |
| 1 | 90,019,450 | 90,039,163 | *ABCC10* | × | × | - |
| 1 | 90,889,627 | 90,939,342 | *CDC5L* | × | × | - |
| 1 | 92,538,026 | 92,807,355 | *RCAN2* | × | × | - |
| 1 | 92,947,771 | 92,975,260 | *SLC25A27* | × | × | - |
| 1 | 93,485,324 | 93,549,929 | *TNFRSF21* | × | × | - |
| 1 | 94,047,591 | 94,230,349 | *PTCHD4* | × | × | - |
| 1 | 97,738,076 | 98,213,846 | *PKHD1* | × | × | - |
| 1 | 98,435,287 | 98,530,078 | *PAQR8* | × | × | - |
| 1 | 98,538,717 | 98,606,632 | *EFHC1* | × | × | - |
| 1 | 98,608,048 | 98,690,116 | *TRAM2* | × | × | - |
| 1 | 99,481,153 | 99,529,420 | *GCLC* | × | × | - |
| 1 | 102,308,800 | 102,780,046 | *DST* | × | × | - |
| 1 | 102,779,984 | 102,831,149 | *BEND6* | × | × | - |
| 1 | 102,881,613 | 102,986,587 | *ZNF451* | × | × | - |
| 1 | 105,941,472 | 107,593,901 | *EYS* | × | × | - |
| 1 | 111,863,825 | 111,957,675 | *COL9A1* | × | × | - |
| 1 | 118,914,596 | 118,980,320 | *ITPR3* | × | × | - |
| 1 | 119,108,583 | 119,140,172 | *SYNGAP1* | × | × | - |
| 1 | 119,144,037 | 119,149,122 | *PHF1* | × | × | - |
| 1 | 119,150,291 | 119,163,372 | *KIFC1* | × | × | - |
| 1 | 119,225,004 | 119,232,375 | *RGL2* | × | × | - |
| 1 | 119,234,730 | 119,243,361 | *WDR46* | × | × | - |
| 1 | 119,252,887 | 119,270,337 | *VPS52* | × | × | - |
| 1 | 130,411,883 | 130,540,455 | *RREB1* | × | × | - |
| 1 | 130,567,273 | 130,593,210 | *SSR1* | × | × | - |
| 1 | 130,604,760 | 130,654,200 | *CAGE1* | × | × | - |
| 1 | 130,654,361 | 130,684,189 | *RIOK1* | × | × | - |
| 1 | 139,672,472 | 139,682,922 | *RBM24* | × | × | - |
| 1 | 196,446,677 | 196,485,687 | *ERAP2* | × | × | - |
| 1 | 204,344,646 | 204,412,936 | *TMEM161B* | × | × | - |
| 2 | 31,155,943 | 31,198,032 | *ZMPSTE24* | × | - | × |
| 2 | 31,204,571 | 31,220,042 | *COL9A2* | × | - | × |
| 2 | 31,245,662 | 31,291,628 | *SMAP2* | × | - | × |
| 2 | 31,311,406 | 31,323,759 | *ZFP69B* | × | - | × |
| 2 | 31,331,710 | 31,347,702 | *ZFP69* | × | - | × |
| 2 | 83,029,941 | 83,871,891 | *DPYD* | × | × | - |
| 4 | 159,411,288 | 159,417,539 | *MTERF1* | × | × | - |
| 4 | 159,456,853 | 159,643,617 | *AKAP9* | × | × | - |
| 4 | 161,975,512 | 162,042,151 | *SGCE* | × | × | - |
| 4 | 162,042,321 | 162,055,735 | *PEG10* | × | × | - |
| 4 | 162,189,534 | 162,488,748 | *PPP1R9A* | × | × | - |
| 6 | 68,905,894 | 69,012,447 | *XRN1* | × | × | - |
| 6 | 69,013,813 | 69,112,247 | *ATR* | × | × | - |
| 6 | 69,132,416 | 69,249,204 | *PLS1* | × | × | - |
| 6 | 78,902,359 | 78,908,907 | *RAP2B* | × | × | - |
| 6 | 80,720,939 | 80,724,530 | *STRIT1* | × | × | - |
| 6 | 89,742,112 | 89,844,449 | *SI* | × | × | - |
| 6 | 89,945,457 | 89,954,703 | *SLITRK3* | × | × | - |
| 6 | 102,085,063 | 102,324,062 | *KCNMB2* | × | × | - |
| 11 | 95,900,902 | 96,246,050 | *CAMK2D* | × | × | - |
| 11 | 96,349,273 | 97,004,412 | *ANK2* | × | × | - |
| 11 | 128,758,412 | 128,859,951 | *RXFP1* | × | × | - |
| 12 | 75,732,267 | 75,951,858 | *CPNE8* | × | × | - |
| 13 | 53,427,389 | 53,468,830 | *KLHL28* | × | × | - |
| 13 | 111,405,001 | 112,004,404 | *UNC13C* | × | - | × |

^a^Chromosome number

^b^Base pair

^c^Detected

^d^Not detected

**Supplementary Table 4.** Identified candidate genes located within the putatively selected regions overlapping between tests within VP2 ELISA study.

| Chr^a^ | Start position (bp^b^) | End position (bp) | Gene ID | Z_(FST)_ | log_2(θπ ratios)_ | XP-EHH |
| --- | --- | --- | --- | --- | --- | --- |
| 1 | 75,914,369 | 76,914,219 | *NKAIN2* | ×^c^ | × | -^d^ |
| 1 | 92,538,026 | 92,807,355 | *RCAN2* | × | × | - |
| 1 | 94,047,591 | 94,230,349 | *PTCHD4* | × | × | - |
| 1 | 97,738,076 | 98,213,846 | *PKHD1* | × | × | - |
| 1 | 105,941,472 | 107,593,901 | *EYS* | × | × | - |
| 1 | 118,914,596 | 118,980,320 | *ITPR3* | × | × | - |
| 1 | 118,998,561 | 119,012,034 | *BAK1* | × | × | - |
| 1 | 119,104,804 | 119,107,733 | *ZBTB9* | × | × | - |
| 1 | 119,108,583 | 119,140,172 | *SYNGAP1* | × | × | - |
| 1 | 119,142,353 | 119,143,935 | *CUTA* | × | × | - |
| 1 | 119,144,037 | 119,149,122 | *PHF1* | × | × | - |
| 1 | 119,150,291 | 119,163,372 | *KIFC1* | × | × | - |
| 1 | 119,199,258 | 119,199,500 | *SMIM40* | × | × | - |
| 1 | 119,204,992 | 119,209,483 | *DAXX* | × | × | - |
| 1 | 119,210,177 | 119,213,508 | *ZBTB22* | × | × | - |
| 1 | 119,225,004 | 119,232,375 | *RGL2* | × | × | - |
| 1 | 119,233,337 | 119,234,649 | *PFDN6* | × | × | - |
| 1 | 119,234,730 | 119,243,361 | *WDR46* | × | × | - |
| 1 | 119,243,652 | 119,245,334 | *B3GALT4* | × | × | - |
| 1 | 119,246,062 | 119,252,735 | *RPS18* | × | × | - |
| 1 | 119,252,887 | 119,270,337 | *VPS52* | × | × | - |
| 1 | 119,293,422 | 119,297,406 | *RING1* | × | × | - |
| 1 | 119,299,016 | 119,301,311 | *HSD17B8* | × | × | - |
| 1 | 289,083,212 | 289,259,797 | *SPEF2* | × | × | - |
| 2 | 83,029,941 | 83,871,891 | *DPYD* | × | × | - |
| 2 | 210,499,604 | 210,592,456 | *R3HCC1L* | × | × | - |
| 2 | 210,595,231 | 210,614,490 | *LOXL4* | × | × | - |
| 2 | 237,782,992 | 237,868,347 | *C2H10orf143* | × | - | × |
| 2 | 237,884,270 | 237,918,382 | *GLRX3* | × | - | × |
| 5 | 136,533,697 | 136,816,788 | *MYCBP2* | × | × | - |
| 6 | 37,748,501 | 38,827,099 | *CADM2* | × | × | - |
| 6 | 40,554,251 | 40,558,056 | *CGGBP1* | × | × | - |
| 6 | 40,558,281 | 40,640,279 | *ZNF654* | × | × | - |
| 6 | 40,644,230 | 40,654,566 | *C6H3orf38* | × | × | - |
| 6 | 69,739,577 | 70,425,604 | *SLC9A9* | × | × | - |
| 6 | 116,025,781 | 116,027,427 | *CPN2* | × | × | - |
| 6 | 116,040,015 | 116,041,775 | *LRRC15* | × | × | - |
| 6 | 116,083,861 | 116,090,578 | *GP5* | × | × | - |
| 6 | 116,089,406 | 116,181,494 | *ATP13A3* | × | × | - |
| 8 | 58,028,494 | 58,075,247 | *LONRF2* | × | × | - |
| 8 | 58,106,135 | 58,138,712 | *CHST10* | × | × | - |
| 8 | 113,418,493 | 113,474,676 | *ATL2* | × | × | - |
| 9 | 97,712,221 | 97,758,164 | *IPPK* | - | × | × |
| 9 | 97,789,108 | 97,832,330 | *BICD2* | - | × | × |
| 11 | 115,732,693 | 116,968,169 | *CCSER1* | × | × | - |
| 11 | 148,947,753 | 148,988,407 | *SMARCA5* | × | × | - |
| 11 | 149,036,912 | 149,163,525 | *GAB1* | × | × | - |
| 11 | 149,249,105 | 149,286,304 | *USP38* | × | × | - |

^a^Chromosome number

^b^Base pair

^c^Detected by the test

^d^Not detected by the test

**Supplementary Table 5.** Identified candidate genes located within the putatively selected regions overlapping between tests within AMDVG ELISA study.

| Chr^a^ | Start position (bp^b^) | End position (bp) | Gene ID | Z_(FST)_ | log_2(θπ ratios)_ | XP-EHH |
| --- | --- | --- | --- | --- | --- | --- |
| 1 | 114,051,167 | 114,583,189 | *KCNQ5* | ×^c^ | × | -^d^ |
| 1 | 119,589,865 | 119,602,944 | *TAP2* | × | × | - |
| 1 | 133,815,924 | 133,823,812 | *C1H6orf52* | × | × | - |
| 1 | 133,829,248 | 133,844,605 | *PAK1IP1* | × | × | - |
| 1 | 133,868,259 | 133,924,333 | *MAK* | × | × | - |
| 1 | 139,672,472 | 139,682,922 | *RBM24* | × | × | - |
| 1 | 139,757,819 | 139,896,722 | *CAP2* | × | × | - |
| 2 | 37,490,288 | 37,492,857 | *FOXD2* | - | × | × |
| 2 | 85,728,541 | 85,788,787 | *SLC35A3* | × | × | - |
| 2 | 85,798,629 | 85,840,447 | *MFSD14A* | × | × | - |
| 3 | 78,449,241 | 78,480,559 | *FRZB* | - | × | × |
| 3 | 78,538,470 | 78,641,444 | *NCKAP1* | - | × | × |
| 3 | 78,665,748 | 78,684,593 | *DUSP19* | - | × | × |
| 3 | 78,707,774 | 78,743,505 | *NUP35* | - | × | × |
| 3 | 103,603,706 | 103,605,271 | *C1QL2* | × | × | - |
| 3 | 158,985,755 | 159,307,870 | *KIAA1328* | × | × | - |
| 3 | 159,307,993 | 159,368,558 | *TPGS2* | × | × | - |
| 3 | 159,377,144 | 159,833,881 | *FHOD3* | × | × | - |
| 5 | 16,354,263 | 16,462,697 | *EFCAB13* | × | - | × |
| 5 | 16,476,392 | 16,527,526 | *ITGB3* | × | - | × |
| 5 | 136,533,697 | 136,816,788 | *MYCBP2* | × | × | - |
| 6 | 40,554,251 | 40,558,056 | *CGGBP1* | × | × | - |
| 6 | 40,558,281 | 40,640,279 | *ZNF654* | × | × | - |
| 6 | 40,644,230 | 40,654,566 | *C6H3orf38* | × | × | - |
| 6 | 75,310,943 | 75,329,636 | *TM4SF18* | × | × | - |
| 6 | 116,089,406 | 116,181,494 | *ATP13A3* | × | × | - |
| 6 | 37,748,501 | 38,827,099 | *CADM2* | × | × | - |
| 7 | 157,927,050 | 158,411,542 | *SBF2* | × | × | - |
| 9 | 97,342,783 | 97,371,444 | *ZNF484* | - | × | × |
| 9 | 97,380,004 | 97,449,092 | *IARS1* | - | × | × |
| 9 | 97,451,038 | 97,474,363 | *NOL8* | - | × | × |
| 9 | 97,476,759 | 97,554,142 | *OGN* | - | × | × |
| 9 | 97,561,154 | 97,571,560 | *OMD* | - | × | × |
| 9 | 97,588,850 | 97,613,706 | *ASPN* | - | × | × |
| 9 | 97,632,133 | 97,665,257 | *ECM2* | - | × | × |
| 9 | 97,712,221 | 97,758,164 | *IPPK* | - | × | × |
| 9 | 97,789,108 | 97,832,330 | *BICD2* | - | × | × |
| 11 | 115,732,693 | 116,968,169 | *CCSER1* | × | × | - |
| 11 | 149,036,912 | 149,163,525 | *GAB1* | × | × | - |

^a^Chromosome number

^b^Base pair

^c^Detected by the test

^d^Not detected by the test

**Supplementary Table 6.** Functional enrichment of candidate genes detected by all three conducted signatures of selection studies.

| Tool | Source | Term | Description | FDR value^a^ |
| --- | --- | --- | --- | --- |
| PANTHER | GO:MF^b^ | GO:0005515 | protein binding | 0.000 |
| g:GOSt | GO:CC^c^ | GO:0005593 | FACIT collagen trimer | 0.019 |
| g:GOSt | GO:CC | GO:0005594 | collagen type IX trimer | 0.019 |
| g:GOSt | GO:BP^d^ | GO:0006942 | regulation of striated muscle contraction | 0.042 |
| g:GOSt | GO:BP | GO:0019932 | second-messenger-mediated signaling | 0.042 |
| g:GOSt | GO:BP | GO:0055085 | transmembrane transport | 0.042 |
| g:GOSt | GO:BP | GO:0060306 | regulation of membrane repolarization | 0.042 |
| g:GOSt | GO:BP | GO:0060307 | regulation of ventricular cardiac muscle cell membrane repolarization | 0.042 |
| g:GOSt | GO:BP | GO:0061337 | cardiac conduction | 0.042 |
| g:GOSt | GO:BP | GO:0070296 | sarcoplasmic reticulum calcium ion transport | 0.042 |
| g:GOSt | GO:BP | GO:0086004 | regulation of cardiac muscle cell contraction | 0.042 |
| g:GOSt | GO:BP | GO:0099622 | cardiac muscle cell membrane repolarization | 0.042 |
| g:GOSt | GO:BP | GO:0099623 | regulation of cardiac muscle cell membrane repolarization | 0.042 |
| g:GOSt | GO:BP | GO:0099625 | ventricular cardiac muscle cell membrane repolarization | 0.042 |
| g:GOSt | GO:BP | GO:1903115 | regulation of actin filament-based movement | 0.042 |
| g:GOSt | GO:BP | GO:1990036 | calcium ion import into sarcoplasmic reticulum | 0.042 |
| g:GOSt | GO:BP | GO:1901894 | regulation of ATPase-coupled calcium transmembrane transporter activity | 0.046 |

^a^False discovery rate adjusted p-value

^b^Gene Ontology molecular function

^c^Gene Ontology cellular component

^d^Gene Ontology biologic process

**Supplementary Figures**


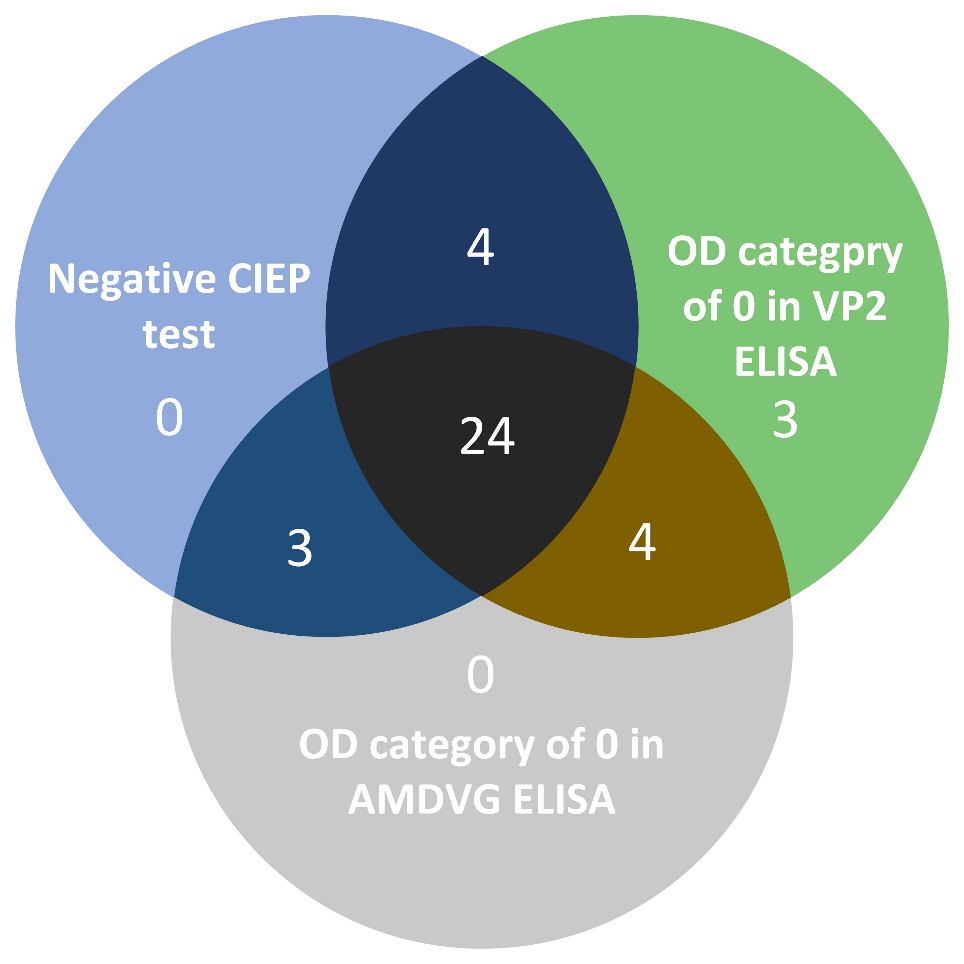


**Supplementary Figure 1.** Venn diagram of overlaps among control groups of the three signatures of selection studies.


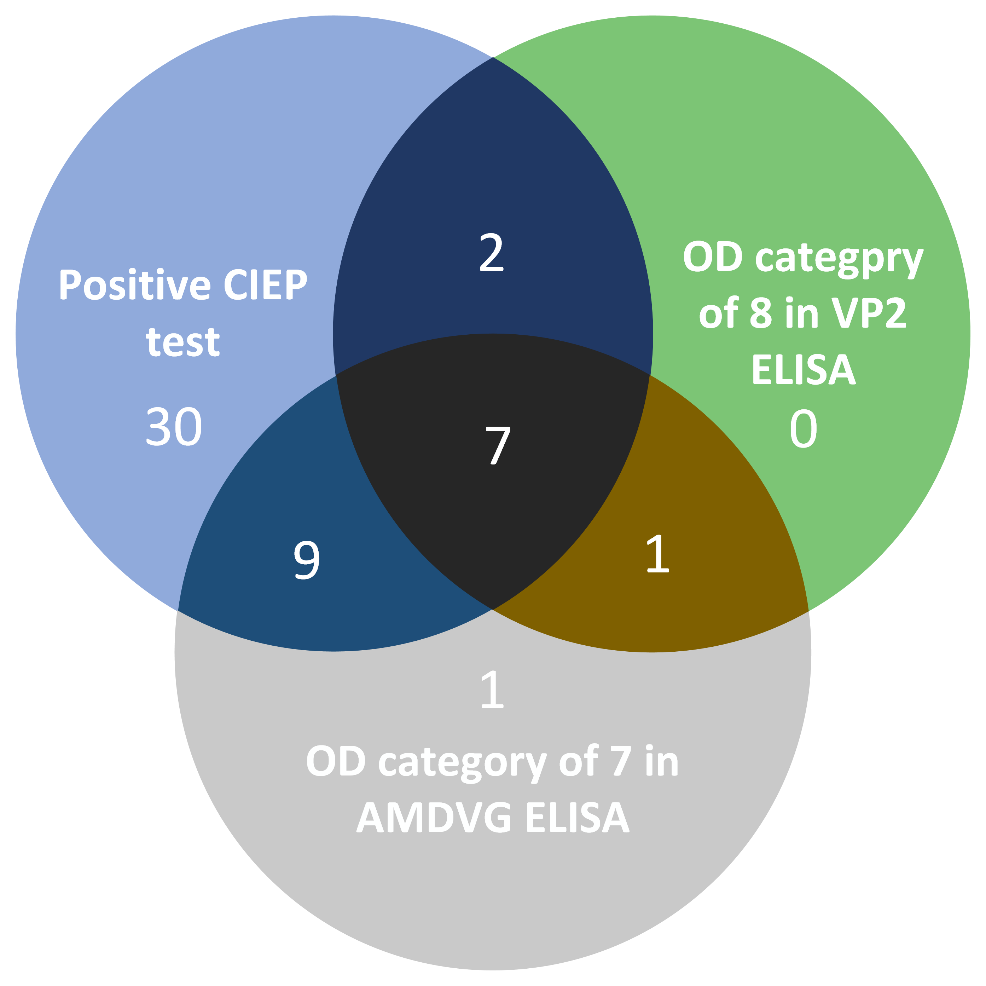


**Supplementary Figure 2.** Venn diagram of overlaps among case groups of the three signatures of selection studies.


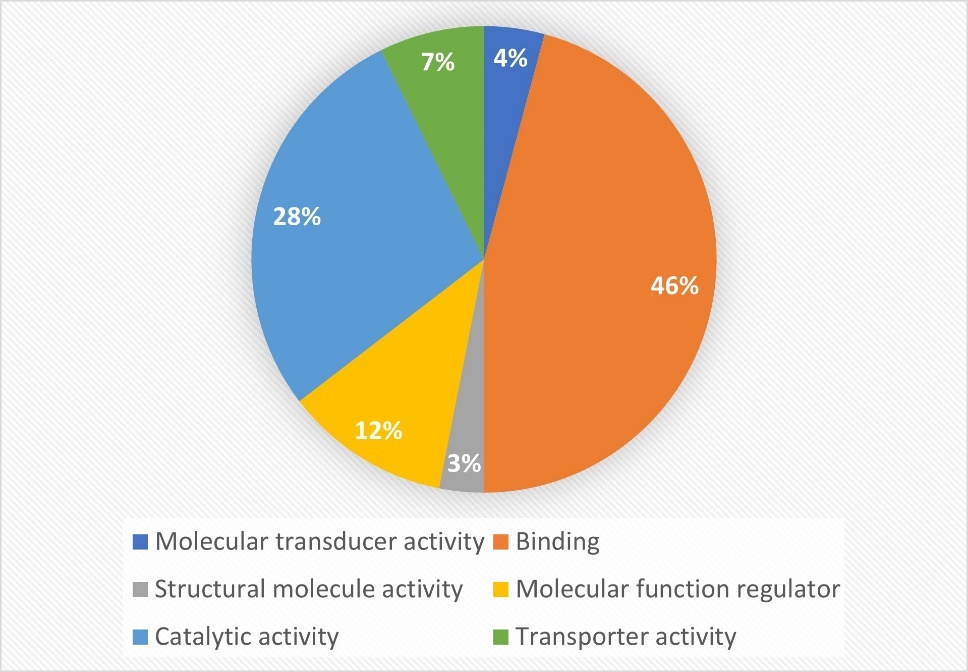


**Supplementary Figure 3.** Pie chart of functional classification of candidate genes detected by all three conducted signatures of selection studies.
